# Green marketing orientation impact on business performance: Case of pharmaceutical industry of Pakistan

**DOI:** 10.3389/fpsyg.2022.940278

**Published:** 2022-09-27

**Authors:** Fatima Shaukat, Jia Ming

**Affiliations:** School of Management, Northwestern Polytechnical University, Xian, China

**Keywords:** green marketing orientation, corporate social responsibility, environmental culture, sustainable competitive advantage, business performance

## Abstract

This study is based on the natural resource based view, which examines the impact of holistic marketing orientation on business performance (BP) by defining the role of enablers and mediators. The drivers, including corporate social responsibility (CSR) and environmental culture (EC) influence, are tested by analyzing the role of sustainable competitive advantage (SCA) as a mediator. The analysis is based on 298 samples collected from top and middle-level managers working in the pharmaceutical industry. Structural equation modeling was undertaken using Smart PLS 3.2.8. The research outcomes reveal that corporate social responsibility and environmental culture have a substantial impact on green marketing orientation (GMO). The results show that GMO has a significant direct and indirect impact on business performance while a full mediation of sustainable competitive advantage exists between the green marketing orientation and business performance. The outcomes will facilitate managers in green marketing strategy and decision making in the long-term, with 3-fold benefits in addition to strengthening their competitiveness.

## Introduction

The spread of the COVID-19 pandemic has impacted the economy globally. The significant negative impact of COVID-19 is undeniable in any industry and its influence varies across different organizations based on their size, type, and sector. The lockdown measures, half attendance rule, and work-from-home safety policies employed to restrict the spread of COVID-19 have negatively impacted 80% of the economy at large. Consequently, many industries are still experiencing the effects and aftershocks of COVID-19. The manufacturing industry in developed countries such as the USA and Germany have suffered great losses and are still vulnerable due to the pandemic. Nevertheless, the manufacturing industries in developing countries like China, India, Pakistan, and Bangladesh are more affected. According to a recent survey, the manufacturing sector of Pakistan is experiencing a 30–50% decrease in revenue (UNIDO, 2021). The government-led COVID-19 vaccination campaign resulted in the vaccination of the maximum number of people within a short time, which has played an integral part in recovering the industrial sector and thus the whole economy. Hence, environmental marketing strategies are essential for the timely recovery of businesses and for enhancing their performance after a crisis.

In the present uncertain era, traditional marketing practices are no longer enough to address changing and uncertain developing markets (Vilkaite-Vaitone and Skackauskiene, 2020). The current patterns of consumption, production, and development are not meeting sustainable industry standards. The business practices of these companies are therefore a major concern and a direct threat to the environment (Calvo-Porral, 2019). The necessity of sustainability in people, product, and service performance demands that organizations should operationalize green marketing throughout their organizational process (Unruh and Ettenson, 2010; Papadas et al., 2017). Companies are encouraged and compelled by researchers, policy makers, government bodies, and business leaders to fulfill their responsibilities toward society by adopting sustainability and good business practices which can help in achieving sustainability along with their business goals (Polonsky, 2011; Geels et al., 2015). According to the World Economic Forum, environmental degradation is a major, worldwide issue that needs to be addressed, and the significance of environmental orientation is increasing (Ip, 2019). The three goals of sustainability (social, economic, and environmental) are both competing and interrelated (Van der Byl and Slawinski, 2015; Hahn et al., 2018). These goals are, in general, considered by organizations only in situations where they bring long-term increases in profit along with the organizational responsibility toward the environment (Caiado et al., 2019; López-Torres et al., 2019). On the other hand, the organizational adoption trend (hesitancy vs. reluctance) toward green marketing orientation for sustainability (Papadas et al., 2017) will increase the organizational risks and losses by not pursuing sustainable business practices. Still, many organizations believe that their competitiveness may reduce by adopting environmentally friendly practices and opting for sustainable development (Nidumolu et al., 2009). In this regard, investigation with empirical evidence is required to rectify this misconception which will help the managers to overcome their resistance to adopting green marketing as a strategy for their sustainable development and competitiveness.

Organizations are working to find methods for improving their relationship with the natural environment. According to the natural resource based view, as the environmental pressure increases, it compels organizations to allocate their resources, abilities, and skills to address the influence of organizational actions and processes with reference to the natural environment, and thereby transform their threats into competitive advantages (Hart and Dowell, 2011; Fraj et al., 2013). The organizational and environmental objectives are guided by GMO in satisfying customer gratification (Papadas et al., 2017). The green marketing orientation is defined as “the recognition of the importance of environmental issues faced by the firms” (Banerjee, 2003). Researchers suggest that GMO can facilitate and empower organizations to develop distinctive capabilities and competencies required to fulfill environmental goals (Crittenden et al., 2011; Chen et al., 2015). The importance of GMO toward policy making nurtures questions including “What factors prompt organizations to implement GMO? Can GMO increase business performance?” Existing literature indicates that GMO and their role in organizations have helped scholars in identifying the gaps that need to be filled.

First, many researchers focused their studies on the link between corporate social responsibility (CSR) and financial performance (Margolis and Walsh, 2003; Orlitzky et al., 2003; Albertini, 2013; Papadas et al., 2019) without considering the impact of environmental strategy and sustainable development empirically (Van der Byl and Slawinski, 2015; Hahn et al., 2018; Caiado et al., 2019). The relationship between CSR and business performance is complicated and requires more attention and exploration. The present study is focused on how CSR helps in the implementation of green marketing orientation and consequently how it impacts business performance through sustainable competitive advantage.

Second, sustainability can't be delivered without changing society's attitude, hence the expected outcomes cannot be achieved (Marshall et al., 2015; Caiado et al., 2019; López-Torres et al., 2019). The organizational culture can nurture or delay the shift toward sustainability (Sroufe, 2017). The implementation of GM is facilitated by strong EC (Fraj et al., 2011). In this regard, incorporated and unified environmental values of organizational culture positively facilitate the effect of GMO on business performance and environmental performance (Moreton et al., 2005). The transformation from traditional to sustainable business operations requires significant and extensive internal modifications that can be supported and reinforced by organizational culture (Epstein and Buhovac, 2014; Wijethilake and Lama, 2019). EC has unquestionable importance and significance in supporting sustainability in organizations (Yang et al., 2017), however, the present literature is insufficient and has rarely discussed empirically its role in implementing a GMO (Wijethilake et al., 2021).

Third, the significance of environmental strategy in yielding competitive advantage and profitability in the long term has been addressed in many studies (Leonidou et al., 2013) but few empirical studies exist regarding the green marketing strategy and SCA relationship (Papadas et al., 2019). Surprisingly, very few studies examined sustainable competitive advantages (Leonidou and Leonidou, 2011). Environmental/green marketing has been an increasing focus for researchers during the last few decades and is now characterized as a key concept in literature on marketing/management (e.g., Chamorro et al., 2009; Polonsky, 2011; Dangelico and Vocalelli, 2017). Literature suggests that organizational value is incremented by environmental strategy but it needs to be integrated into the corporate strategy to achieve the obligations toward sustainability (Polonsky, 1995; Porter and Van Der Linde, 1995; Menon and Menon, 1997; Banerjee, 2003). Similarly, the significant value of an environmental strategy and benefits in the longer term have been stressed by various studies that suggest it may add profitability and a strong competitive advantage to organizations (e.g., Leonidou et al., 2013). Despite these research streams on environmental strategy, the empirical literature about the relationship between contemporary green marketing strategy and the competitiveness of firms is still very limited. The link between environmental/green marketing and business performance is, however, identified in several research studies (e.g., Miles and Covin, 2000; Baker and Sinkula, 2005), yet the environmentally driven competitive advantage (which is a strategic, long-term objective) has been addressed by few studies (Leonidou and Leonidou, 2011). Hence a comprehensive study is required to examine the competitive advantage under the strategic green marketing approach, which establishes a significant research gap and an opportunity for future research. The present study will increase the knowledge base by empirically proving the mediation of a green marketing orientation (GMO) on business performance (BP) through sustainable competitive advantage (SCA) and the dual positive impact of GMO on SCA and BP.

Fourth, there is limited research on GMO in the context of the pharmaceutical industry of Pakistan, regarded as a developing country. Researchers stress that a GMO is a vital and long-term tool for SCA while confronting an ever changing and dynamic market. The green marketing orientation is a holistic approach that acts as the backbone for organizational transformation and achieving sustainable competitive advantage. Moreover, recent studies highlight the impact of a GMO on marketing performance and financial performance in manufacturing industries which emphasizes that more studies should be conducted for different industries to understand this phenomenon completely (Papadas et al., 2017, 2019). Industries in developing countries like Pakistan operate differently as compared to developed countries, therefore they need to be explored separately. Pakistan is one of those developing countries whose local pharmaceutical industry is growing tremendously (Aamir and Zaman, 2011) while producing around 70–80% of generic and branded generics for the country's needs (Aamir and Zaman, 2011; Atif et al., 2017). Pakistan legislated an environmental protection act in 1997 to cope with environmental issues and problems in the country. Although there is a specific Environmental Act for protecting the environment in Pakistan, the government of Pakistan is not taking serious initiatives to enforce actions or motivate organizations to adopt environmental policies; the regulatory framework is not working properly. In this context, the present research is focused on pharmaceutical manufacturing organizations to explore the impact of green marketing orientation on business performance in this specific sector or industry in a different context (Papadas et al., 2017). Similarly, Fatoki (2019) mentioned that the impact of green marketing orientation on business performance should be examined by using different measures. A gap, therefore, exists for a thorough investigation of the intersection between holistic green marketing and its impact on business performance through sustainable competitive advantage, as well as the factors supporting the green marketing strategy at the corporate level.

The main purpose of this study is to confirm whether corporate social responsibility (CSR) and environmental culture (EC) have any impact on the implementation of green marketing orientation (GMO) in the pharmaceutical industry; and then to determine any impact on business performance (BP). Second, the study aims to verify the mediating impact of sustainable competitive advantage (SCA) between green marketing orientation (GMO) and business performance (BP).

Based on identified gaps, the following research questions are proposed for the present study:

***RQ1***. Do CSR and EC have any impact on GMO in the pharmaceutical industry of Pakistan?
***RQ2***. Is there a direct impact of GMO on BP in the pharmaceutical industry of Pakistan?
***RQ3***. Does SCA mediate the relationship between GMO processes and BP in the pharmaceutical industry of Pakistan?

The first section of the paper is comprised of hypothesis development and research framework; then the research methodology is presented which includes sampling, data collection measures, and data analysis procedures. Third, data analysis and results are discussed; last, the discussion, conclusion, implications, limitations, and future research directions are presented.

## Literature review and hypothesis development

The theoretical framework presented in the present research is based upon the Green Marketing Orientation (GMO) theory (Papadas et al., 2017) and the theoretical concepts of organizational culture, business performance, corporate social responsibility, and competitive advantage. In this context, the present research addresses the corporate-wide aspects of the green marketing concept. Moreover, a holistic green marketing strategy has been adopted to capture the organizational modern strategic and internal initiatives (Menon and Menon, 1997; Banerjee et al., 2002). For this purpose, a brief literature review has been included for conceptualizing the arrangement and interaction of various significant factors.

### Green marketing in pharmaceutical sector of Pakistan

Global climate change, environmental pollution, increased laws for environmental protections, and environmental policies have pressured organizations and companies to pay attention to green management. Consequently, the major concern for firms in the pharmaceutical industry is to achieve sustainability in their business processes (Agar et al., 2014; Amran and Ooi, 2014; Sheldon, 2017; Milanesi et al., 2020). The pharmaceutical industry contributes significantly to the economy at the national and global level, therefore the evolution and development of the pharmaceutical sector contributes a lot to the lives of people by improving their health and wellbeing, and thereby improving their quality of life (European Federation of Pharmaceutical Industries Associations, 2019). Pakistan's standing in the pharmaceutical sector of Asia-Pacific is at 10th position (Atif et al., 2017) with an estimated market size of around $ 13.1 million (Butt, 2021), operating with 647 registered pharmaceutical firms as of 2019 (DRAP, 2019).

The published literature about the pharmaceutical industry of Pakistan however fails to address the link between green marketing orientation and increased business performance with the complementing roles of environmental culture and corporate social responsibility. Additionally, the role of green marketing orientation toward business performance and sustainable competitive advantage needs proper attention. Irrespective of its size in terms of market share, growth potential, and GDP contribution, few studies have been conducted to figure out the mechanism of environmental policy making, implementation, and its contribution toward performance.

### Green marketing orientation

The green marketing orientation is conceptualized as the process management uses in analyzing, identifying, creating, and satisfying consumers' and society's needs in a profitable and sustainable manner by adopting sustainable practices from production to post-purchase services (Peattie, 1995). According to Gordon et al. (2011) the development and commercialization of sustainable goods and services is facilitated by the green marketing orientation. Green marketing helps in filling the gap between traditional marketing practices and environmental and societal realities (Calvo-Porral, 2019). The green marketing orientation is defined by Papadas et al. (2017) as “the extent to which an organization engages in strategic, tactical, and internal processes and activities with an aim to create, communicate, and deliver products and services with the minimal adverse environmental impact”. In this context, organizations should employ a holistic green marketing orientation approach to achieve their desired goals.

### CSR and green marketing orientation

The World Business Council for Sustainable Development defined CSR as “the continuing commitment by business to behave ethically and contribute to economic development while improving the quality of life of the workforce and their families as well as of the local community and society at large” (Dahlsrud, 2008). The top management of an organization has responsibilities toward society to make policies and act in the best interest of society both ethically and morally (Davis, 1973). Industrial experts and academics are paying more attention to corporate social responsibility (Rashid et al., 2014) and its role as the proactive strategic agent motivating corporate environmental behavior (Kärnä et al., 2003). Notably, corporate social responsibility acts as a strategic tool in directing attention toward corporate marketing instead of consumer marketing (Powell et al., 2007). Organizations, therefore, can use their environmentally responsible behavior and actions as marketing tools, as addressed by many researchers (Punitha and Rasdi, 2013). The incorporation of social dimensions led to the concept of “holistic marketing” and stakeholder perspectives of marketing and CSR (Kotler and Keller, 2006). CSR is therefore attracting more attention but at the same time, few studies have examined environmental CSR (Nie et al., 2019).

***H1*. **Corporate social responsibility (CSR) is positively associated with green marketing orientation (GMO).

### Environmental culture- EC and green marketing orientation- GMO

Over time, companies have realized that they cannot isolate themselves from society, their responsibility toward society, nor the community as a whole. They must behave responsibly toward the environment by incorporating environmental objectives in their corporate values and thereby integrating environmental objectives and sustainability as part of their organizational culture. The top management of many organizations has adopted environmental values as part of their organizational culture and, as a result, top management's cultural values have influenced the implementation of environmental values and behaviors (Junsheng et al., 2020).

The sustainable CA serves as one of the key resources to generate an enhanced corporate culture according to the Resource-Based View (RBV) theory (Barney, 1986). Superior corporate culture is therefore represented by corporate environmental ethics for the attainment of sustainable development (Chang, 2011). The concept of green human capital has been introduced by Chen (2008) as the combination of knowledge, skills, capabilities, and innovation of employees for achieving the organizational goals toward green innovation or environmental protection. Moreover, the firms can improve their environmental marketing strategies to enhance their business performance outcomes by developing a strong environmental culture (Fraj et al., 2011).

Organizational culture refers to the norms, values, and assumptions that are shared among the organizational members and that tend to persist over time (Kotter and Heskett, 1992). Environmental Culture measures and incorporates how an organization deals with environmental safety values from all aspects of the organization which is reflected in the organizational mission statement, formal policies, and procedures, and training and information programs for employees and managers (Fraj et al., 2011).

Human activities are influenced by the environment and therefore they revolve around the respective environment. To make environmentally safe strategies, the organizational culture should reflect this from their shared vision, mission, policies, and procedures that add value to learning, and enhance creativity and employee support (Fraj et al., 2013). Pro-environmental strategies and behaviors are influenced by top management. Upper management motivates and spurs green environmental culture to operationalize environmental recognizant policies, programs, reward mechanisms, and appropriate communication while addressing environment related matters.

Steady EC influences the business to gain advantages by applying appropriate practices to ensure that similar environmental values and norms are followed at all organizational levels. To strengthen their environmental positions, companies publicize their efforts by applying and displaying their green marketing orientation. It is time to adopt green strategies for sustainable development from all aspects as environmental problems affect human lives and their activities. To effectively implement strategies, the respective culture plays an important role, therefore, environmental culture in an organization facilitates the formation and implementation of sustainable strategies.

The well-built environmental culture and incorporated environmental aspects are reflected as important organizational resources. This is because the environmental aspects lead to society's wellbeing and improved quality of life. Companies with strong environmental cultures are more prone to an overall green marketing orientation, which acts as a competitive advantage and leads to better business performance.

***H2*. **Environmental culture is positively associated with the implementation of green marketing orientation.

### Green marketing orientation- GMO and business performance- BP

Different definitions of business performance are focused on the effectiveness or success of a business, employee performance, ability to create value for customers, productivity, flexibility and adaptability, achievement of goals, and stakeholder satisfaction. To achieve business goals and objectives; business performance is defined as the potential of any business to gain maximum output by utilizing the available resources effectively.

Currently, businesses are not just concentrating on profits but the betterment of society as well which is done by showing concern for social and environmental issues. Threats of global warming have led the world to develop high concerns for environmental protection. As a result, a new format of business has emerged which is known as Green Business. By providing such goods and services that are environmentally beneficial; businesses are transforming the user trends and usage patterns of consumers. In this manner, a green business assimilates the foundation of sustainability into the business process.

Businesses are following green marketing practices for enhancing their business performance. The concept of green marketing is a corporate goal regarding business profitability and getting better business performance through environmentally sustained practices. Businesses that execute a green marketing orientation give the impression that they are concerned about societal wellbeing and the natural environment. In this manner, GMO acts as an important strategic resource that helps to enhance and strengthen business performance.

***H3*. **Implementation of a green marketing orientation positively increases business performance.

### Green marketing orientation- GMO, sustainable competitive advantage- SCA and business performance- BP

Competitive advantage is considered a long-term strategic objective that typically results in high business profits. In this manner, sustainable competitive advantage refers to value creation in which a firm pursues high innovation by driving market orientation. A business is said to have a sustainable competitive advantage when the present or potential competitors may not imitate the business resources, or it will be costly for them. Some competitive advantages are sustainable if competitors are unable to imitate the source of advantage or if no one conceives a comparatively better offering (Davis, 1973). Sustainable marketing can help companies to adopt a long-term perspective and value continuous profit. According to the resource-based view, the subjects of sustainable competitive advantage are resources and skills (Barney, 1991; Madhani, 2010).

If the business wants to achieve a sustainable competitive advantage, it must integrate environmental thinking into all aspects of marketing (Chaudhary and Batra, 2018). The RBV by Barney (1986, 1991) concluded that valuable business resources and capabilities are important sources of sustainable competitive advantage for a firm. Global competition is now becoming increasingly fierce; therefore, green marketing is considered a strength toward the creation of sustainable competitive advantage.

**Figure 1:**
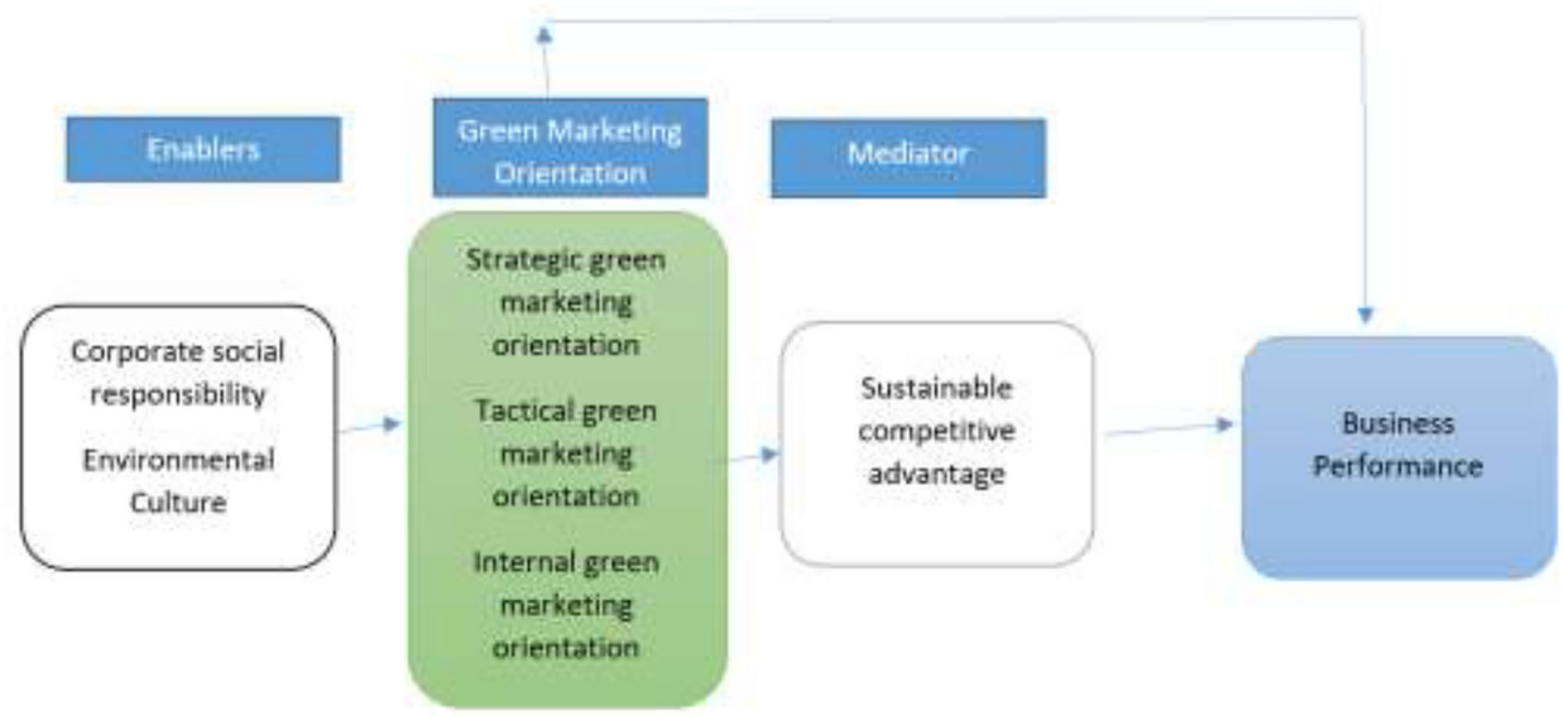
Proposed model.

Business success is deeply rooted in having some sort of competitive edge over competitors. Hence, business strategies are destined to have some competitive advantages besides better performance (Day, 1984; Ulewicz and Blaskova, 2018). Based on previous discussion, conceptual framework is drawn and shown as Figure 1. Businesses in this regard employ various strategies, technologies, and other resources. The competitive advantage serves as a kind of shield against their competitors which empowers them to retain their unique characteristics (Porter, 1989).

The study also addresses a significant knowledge gap and extends our understanding of the green marketing-competitiveness relationship by uniquely highlighting the impact of a strategic green marketing approach on competitive advantage. The study validates the mediating effect of strategic green marketing on business performance (BP) through competitive advantage (CA). Hence the study underpins the dual positive effect of strategic green marketing on CA and BP.

The concept of green marketing has gained significant importance in the last few years. This is because GMO is considered to be a business strategy that allows businesses to make products that are environment-friendly and create value for customers. In this manner, GMO acts as a strategic resource that helps gain sustainable competitive advantages for businesses. Therefore, it is validated that GMO is a firm-specific intangible resource that can help businesses to realize value when marketing pays considerable interest/attention to sustainability.

The NRBV by Hart (1995) suggested that the competitive advantage of a firm is based on its relationship with the respective natural environment where this relationship can be improved by a GMO. Past investigations outline that green products as well as green processes play an important role in achieving CA (Sharma and Vredenburg, 1998; Chen et al., 2006; Leonidou et al., 2015). Similarly, environment-friendly processes result in a sustainable competitive advantage (Papadopoulos et al., 2010; Ghodeswar and Kumar, 2014).

Prior research indicates that a business achieves competitive advantages due to its proactive environmental strategies by implementing rare, unique, and complex capabilities that help the business to differentiate (Hart, 1995; Miles and Covin, 2000) at the organizational level, and its ability to compete sustainably in a global world while being adaptive to changes. Recently, businesses have started to adopt GMO to achieve a competitive advantage and improve business performance, by focusing on their differentiation strategies and satisfying environmentally conscious consumers.

***H4*. **Green marketing orientation has a positive effect on business performance through sustainable competitive advantage.

## Research methodology

### Setting

The economic backbone of a country is its industrial sector but, at the same time, the hazardous emissions and effluent from industries are destroying the environment (Mahmood et al., 2019). Pakistan Environmental Regulatory Agency has poor control in this regard despite having a policy framework and regulatory structure. During the past few years, no considerable action has been taken toward controlling the environmental damage from the industrial sector. The poor air quality in Pakistan demands serious attention since it is damaging the health of millions of people on a daily basis. One of the biggest cities in Pakistan, Lahore, has the worst air quality index according to a study conducted in the year 2021, consequently, the city was ranked as the second worst in the world (Butt, 2021). The air quality is also becoming harmful in other cities including Peshawar, Karachi, Multan, and Faisalabad.

### Sample and data collection

Data were collected from different types of organizations (domestic and multinational) and from different levels of organizational managers working in the pharmaceutical industry to assure triangulation in data sources (Turner and Turner, 2009). Moreover, the participants of this study included managers from the organizations usually ranked as top or middle managers, such as chief executive officers, branch managers, divisional managers, marketing managers, and operations managers. This is because the top and middle managers are usually involved in the policy making and implementation although, their designation or rank titles vary in different companies according to the size and type of organizations. Data were collected between July and December 2021. In addition to physical visits, a questionnaire was shared through emails and the WhatsApp smartphone application. The data were collected through a convenient sampling technique since the targeted population included top and middle level managers who are not easily accessible, as well as social distancing restrictions due to COVID. Almost 900 questionnaires were distributed among the population, of which 337 were returned i.e., a 37% response rate. Among these, 39 were excluded due to incomplete data. Among the remaining questionnaires, 154 were distributed through personal visits, while 102 returned and 03 were discarded. Furthermore, 746 were distributed online (WhatsApp and Email), out of which 235 were returned and 36 were discarded.

The distribution of the questionnaire is represented in Table 1, separated by personal visits and online distribution. The table also reflects the total distributed questionnaire, those returned, and others that were discarded due to them being incomplete or as a result of other issues. The sample size, therefore, meets the requirement of structural equation model (SEM) analysis (Kline, 2011).

**Table 1:** Questionnaire distributed for data collection.

**Questionnaires distributed**	**Distributed**	**Returned**	**Discarded**
Personal visits	154	102	3
Online (WhatsApp & Email)	746	235	36
Total questionnaire	900	337	39

### Measures

The variables used in the study were “Corporate Social Responsibility, Environmental Culture, Green Marketing Orientation, Sustainable Competitive Advantage, and Organizational Performance”. The variables were measured by using a 5-point Likert Scale (1 = strongly disagree, 5 = strongly agree). The corporate social responsibility construct captures the important activities that focused on the protection and fortification of the society, environment, institutions, and future generations whereby measurement items were adapted from Papadas et al. (2019) with seven items. To measure environmental culture, the scale proposed by Banerjee (2002) and employed by Fraj et al. (2011) was used. These scales measure the extent to which an organization embraces and implements environmental values and reinforces them through organizational policies, mission statements, and various programs for the employees. The five-item scale was used for determining responses and the managers were asked to rate the propositions on a 5-point Likert Scale. The green marketing orientation was measured using the scale adapted from Papadas et al. (2017), where the green marketing orientation is used to describe the holistic approach of the organization toward natural environmentalism. For sustainable competitive advantage, a six-item scale developed by Chang (2011) was used (this scale has also been used in many other studies) (Gürlek and Tuna, 2018; Papadas et al., 2019). The organizational performance was measured by a nine-item scale adopted from Burke (1984) by Feder and Weißenberger (2021) while the respondents were asked to compare the performance of the organization compared to competitors, as employed previously in other studies (González-Benito and González-Benito, 2005; Helmig et al., 2016).

## Data analysis procedure

### Partial least squares- PLS tool

For data analysis, the Smart-PLS 2.0 software package was used (Ringle, 2005), The PLS-SEM is the prominent method for analyzing, understanding, and validating the causal relationships among complex variables (Gudergan et al., 2008). It has already been used by many researchers in various social sciences, management, and business studies to handle data in a better manner (Hair et al., 2014). Other research was consulted to assess the causal relationships between variables based on existing theories and involving complex structures (Ali et al., 2018). This statistical tool helped in the explanation of complex relations in the overall model (Valaei et al., 2017). The analysis involves two stages of investigation, i.e., measurement model description and structural model assessment. PLS measures the overall fitness of the model and the discriminant validity through the heterotrait-monotrait ratio of correlations (HTMT) (Henseler et al., 2015) whereby the rigorous procedure suggested by Preacher and Hayes (2008) was used for mediation analysis as it is a more appropriate method with the PEL-SEM procedure. Notably, most recent empirical studies in the GM field have used the PLS-SEM tool for testing data (Chung, 2020).

### Data analysis results

The analysis consisted of two parts, the measurement assessment of data and structural model assessment, as described below.

### Measurement model assessment

The analysis of the measurement model was undertaken in the first phase according to previous studies to confirm the reliability, validity, and dimensions of the model (Hair et al., 1998). As a result, no factors were removed while evaluating the measurement model, because all the factor loadings are above or close to the suggested value of 0.60. Therefore, scales adopted from previous studies were used without any modification. The AVE and CR of all the variables are also matching, with the ideal suggested value of 0.50 and 0.70, respectively (Hulland, 1999), which proved model convergent validity and reliability as shown in Table 2. Discriminant validity is evident in Table 3 (Fornell and Larcker, 1981). The correlation between the two dimensions is greater than the square root of its AVE in each dimension; it validates its discriminant validity (Thorndike, 1995). The internal consistency existing between each dimension is shown by Cronbach alpha values that are larger than 0.7. The cross-loading values shown in the table indicate that the indicator values are higher than their cross-loadings with other constructs. Table 4 shows that the HTMT is below 0.9 which meets its threshold requirement (Gold et al., 2001). All these values indicate the validity and reliability of the construct as shown in Tables 3–5.

**Table 2:** Construct validity.

**Construct**	**rho_A**	**CR**	**AVE**	**Cronbach's Alpha**
BP	0.955	0.961	0.733	0.954
CSR	0.963	0.969	0.816	0.962
EC	0.933	0.946	0.744	0.931
GMO	0.979	0.980	0.704	0.979
IGMO	0.951	0.960	0.773	0.951
SCA	0.910	0.937	0.789	0.910
SGMO	0.954	0.960	0.727	0.953
TGMO	0.920	0.938	0.753	0.917

**Table 3:** Fornell-Larcker criterion.

	**BP**	**CSR**	**EC**	**GMO**	**IGMO**	**SCA**	**SGMO**	**TGMO**
BP	0.856							
CSR	0.769	0.903						
EC	0.800	0.823	0.862					
GMO	0.846	0.838	0.710	0.839				
IGMO	0.791	0.821	0.863	0.769	0.879			
SCA	0.850	0.821	0.837	0.705	0.878	0.888		
SGMO	0.835	0.810	0.902	0.671	0.814	0.868	0.853	
TGMO	0.834	0.806	0.880	0.671	0.837	0.815	0.712	0.868

**Table 4:** Results of proposed.

**Relations**	**Path coefficient**	**Sample mean (M)**	**Standard deviation (STDEV)**	***T*-value**	***P*-value**	**Decision**	**Model fit**
CSR -> GMO	0.954	0.903	0.069	7.456	0.003	Supported	SRMR = 0.074
EC -> GMO	0.966	0.965	0.062	15.557	0.000	Supported	RMS_theta = 0.225
GMO -> BP	0.372	0.373	0.127	4.909	0.004	Supported	
GMO -> IGMO	0.969	0.969	0.004	220.802	0.000	Supported	
GMO -> SCA	0.905	0.905	0.019	46.700	0.000	Supported	
GMO -> SGMO	0.971	0.971	0.005	180.971	0.000	Supported	
GMO -> TGMO	0.971	0.971	0.005	180.380	0.000	Supported	
SCA -> BP	0.523	0.522	0.123	4.241	0.000	Supported	
BP	*r*^2^ = 0.764		GMO->BP			f2 = 0.107	
GMO	*r*^2^ = 0.829		SCA–> BP			f2 = 0.209	
SCA	*r*^2^ = 0.939		CSR–> GMO			f2 = 0.003	
			EC–> GMO			f2 = 0.745	

**Table 5:** Summary of mediation result.

**Total effect (GMO -**> **BP)**		**Direct effect GMO-**> **BP**			**Indirect effect of GMO–**>**BP**			
**Coefficient**	***p*-value**	**Coefficient**	***p*-value**		**Coefficient**	**SD**	***T*-value**	***p*-value**
0.845	0.00	0.372	0.004	GMO->SCA-> BP	0.437	0.115	4.13	0.00

### Structural model assessment

The overall model fits (SRMR = 0.074; RMS_theta = 0.225). Analysis showed R^2^ values of 0.764, 0.829, and 0.939 for BP, GMO and SCA, respectively. The construct in the model predictive power is supported by R^2^ values (Sarstedt et al., 2014) as it is above the required value of 0.10 (Falk and Miller, 1992). Effect f2 values were then used to predict the extent of contribution of exogenous variables toward endogenous R^2^ values. In the present study, the GMO was predicted by CSR and EC, while SCA was predicted by GMO, and BP by SCA. The evaluation of the measurement model was undertaken during the second phase and the hypothesis was tested. First, the direct impact of CSR and EC was analyzed on GMO. Second, the direct impact of GMO and BP was measured. Then the direct impact of SCA on BP was tested. Later the bootstrap resampling technique was used with 5,000 resamples to analyze the significance of direct paths (Ringle, 2005). The test results of the hypothesis for direct relations are summarized in Table 3.

The overall model fits (SRMR = 0.074; RMS_theta = 0.225). Analysis shows R^2^ values of 0.764, 0.829, and 0.939 for BP, GMO, and SCA, respectively. The construct in model predictive power is supported by R^2^ values (Sarstedt et al., 2014) as it is above the required value of 0.10 (Falk and Miller, 1992). The effect values f2 were used to predict the extent of the contribution of exogenous variables toward endogenous R^2^ values. The GMO was predicted by CSR and EC while SCA was predicted by GMO, and BP was predicted by SCA. During the second phase, the evaluation of the measurement model was undertaken, and the hypothesis was tested. First, the direct impact of CSR and EC was analyzed on GMO. Second, the direct impact of GMO and BP were measured. Then the direct impact of SCA on BP was tested. Later the bootstrap resampling technique was used with 5,000 resamples to analyze the significance of direct paths (Ringle, 2005). The test results of the hypothesis for direct relations are summarized in Table 3.

**Figure 2:**
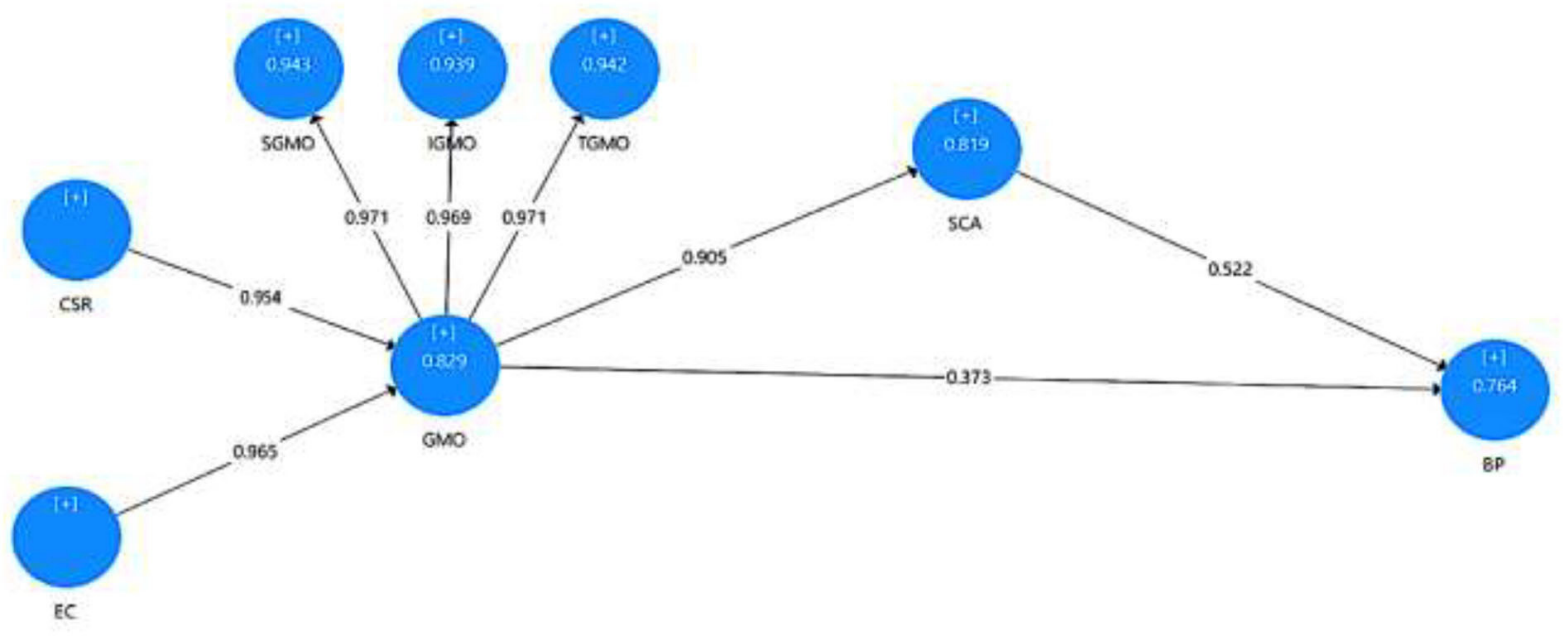
Hypothesized model.

The research results indicated, as shown in Figure 2, that CSR has a substantial positive influence on GMO (β = 0.954, *t* = 7.45, *p* < 0.001) and EC on GMO (β = 0.966, *t* = 15.55, *p* < 0.001). Hence, H1 and H2 are validated.

Similarly, the influence of GMO on BP is significant direct positive (β = 0.372, *t* = 4.909, *p* = 0.004); meaning H3 is accepted.

The impact of GMO on SCA was also found to be significant (β = 0.905, *t* = 46.7, *p* < 0.001); hence, H4 is validated.

Moreover, the significant direct and positive impact of SCA on BP was also validated by research results (β = 0.523, *t* = 6.421, *p* < 0.001), meaning H5 is validated.

Finally, the indirect impact of GMO on BP through SCA was tested using mediation assessment. The result shows a positive influence of CSR and EC on GMO, as shown by Figure 2.

### Mediation effect

The last test for mediation was conducted according to hypotheses where the SCA mediates the relationship between GMO and BP. The results show that the direct impact is (β = 0.372, *t* = 2.931, *p* < 0.004); while the indirect impact is (β = 0.43, *t* = 4.13, *p* < 0.00). The analysis of mediations shows that H5 is supported. Table 5 shows the mediation results.

## Discussion

The importance of sustainability in today's dynamic and competitive market has grown in recent years, especially in the era of pandemics and uncertainty. Our research contributes in three ways: first, it demonstrates the application of green marketing and its impact on sustainable competitive advantages in the context of Pakistan. Second, it validates green marketing from a corporate and stakeholder perspective while incorporating its drivers. Third, it incorporates prior studies and extends to future studies by showing its impact on business performance in the context of the pharmaceutical industry of Pakistan.

The domain of environmental/green marketing strategy and its impact has been addressed by various previous studies with reference to developed countries like China, Greece, and Spain (Li et al., 2018; Papadas et al., 2019) but research is very limited for developing countries like Pakistan. This is because environmental issues are given less attention in these countries while little to no efforts are made toward a regulatory framework at the governmental level, nor toward sustainable growth from direct organizational activities. The environmental/green marketing strategy reserves a significant place in developed countries; therefore, many organizations have separate departments to address this domain under corporate social responsibility. Alternately, in developing countries, these functions are managed by a separate manager. In this context, the present study would attempt to create awareness about environmental policies at an organizational level which may trigger relevant environmental strategies at an organizational level leading to improved business performance and sustainable competitive advantage. The study will further assist and help the managers in understanding and adopting the holistic approach toward green marketing at the corporate level and achieving sustainability.

The study highlights the importance of holistic green marketing for enhancing the business performance of organizations. This research examines the impact of CSR and EC on GMO and the indirect impact on BP through the mediation of SCA within the context of the pharmaceutical industry of Pakistan. Pakistan is a developing country with accelerated growth in the pharmaceutical industry whereby this contextual setting is understudied. In this regard, the first part of the study focuses on the drivers that lead to the positive application of green marketing orientation in organizations and extends past research (Papadas et al., 2019; Chung, 2020) which was carried out on the drivers of green marketing. The enablers of CSR and EC help and support the implementation of environmental strategies within organizations that lead to better profitability and performance. The present study supports the complete integration of environmental policy into organizations instead of only focusing on corporate social responsibility separately. The results of the present study confirm that CSR has a positive impact on GMO and validates the previous studies, while the present study also confirms the positive impact of EC in implementing GMO. Many research studies found that organizational culture plays a vital role in implementing or bringing change in organizations. The attitude of people and their understanding influence business operations to achieve organizational goals.

Additionally, the present study results proved that GMO implementation leads to better performance of an organization which clarifies that the GMO also increases the SCA of an organization and increases the performance of an organization. It therefore confirms and extends the previous knowledge regarding the green marketing strategy as a source of competitiveness. The present research takes a holistic approach to the green marketing strategy and its competitiveness in a developing country within the context of the pharmaceutical industry and addresses the critical research gap. Previously, studies were done in developed countries in the context of manufacturing industries.

The tangible evidence from the sustainable CA is provided to the managers from the empirically-tested conceptual framework, which needs to be enhanced, for adopting a holistic GMO. This is employed for moderating the unresolved tension being perceived by managers between green marketing and the competitiveness of the firm. The study, therefore, facilitates the GM literature by highlighting the contemporary yet unexplored relationship. Past research in this domain reflects the need for a contemporary strategic approach to establish the link between GM and CA whereby previous research was more focused on BP (e.g., Pujari et al., 2003; Fraj-Andrés et al., 2009).

The study also confirms the mediation effect of green marketing orientation on business performance through sustainable competitive advantage as previously stated (Papadas et al., 2019; Chung, 2020). The present study expands on the dual impact of GMO on SCA and business performance. Different variables have been used to measure the performance of an organization including marketing performance, financial performance, and environmental performance, but measuring GMO impact on business performance was still missing. The present study is based on the green marketing theory that states that a green marketing strategy enhances the competitiveness of an organization which in response increases organizational performance in terms of profitability, market share, and customer satisfaction.

## Conclusion

Our findings on the relationship between a GMO and its drivers (CSR, EC) add to the literature on holistic green marketing. The study summarizes that the implementation of environmental strategy is facilitated and supported by CSR and green organizational culture which proves that people's attitudes matter in bringing change. The successful implementation of the environmental strategy has a positive impact on the performance of the organization. Moreover, the role of sustainable competitive advantages between GMO and BP in the pharmaceutical industry was ascertained. While previous studies focused only on the relation of strategic green marketing to financial performance (Papadas et al., 2019), GMO impact on green practices (Li et al., 2018), GMO impact on green image and loyalty (Chung, 2020), the present study, analyzed the relation of holistic green marketing with BP and mediations. The analysis reflected the substantial mediating role of SCA among the GMO and BP.

## Implications

This study provides significant insights and implications for managers and business practitioners. The present study establishes aimed to (a) examine the concept of strategic GM on competitiveness, and (b) empirically test their relationship under the influence of internal GM actions and CSR. In this context, the present study provides a significant contribution toward the development of environmental/green marketing. The study initially extends the findings of previous studies with reference to the drivers of strategic GM. The findings then support a corporate environmental integration approach, which is essential for competitive success rather than undertaking corporate social/environmental responsibility exclusively (Porter and Van Der Linde, 1995; Menon and Menon, 1997). Moreover, the study offers additional support by examining the impact of CSR and EC on GMO for the strategic role of organizational culture and employees shared values in developing a long-term GM strategy.

The application of green marketing as a policy at the corporate and industrial level reflects long-term commitment and investment toward a green economy that can be used as a strategic instrument based on its positive impact on SCA and BP. Incorporating green policies at the corporate level helps organizations differentiate themselves from greenwash-focused competitors—those using superficial green programs for enhancing their corporate image—by implementing green initiatives e.g., using environmentally friendly production methods, disposing of chemical wastes properly, investing in the research and development of medicines, which provides 3-fold benefits to consumers, society, and the company.

Second, the results indicate that CSR and EC positively help in the implementation of GMO. The positive relation of CSR has been checked and validated in previous studies (Papadas et al., 2017, 2019; Chung, 2020) but environmental culture's role as a driver of green marketing has not been measured before. Similarly, researchers previously studied environmental culture as an intangible asset that positively moderates the impact of a green marketing strategy on financial performance (Fraj et al., 2011) but the present research clarifies and suggests how positive environmental culture is as a driver toward implementing the environmental strategy in organizations and how it affects the business performance positively.

Third, the present empirically tested conceptual framework will help managers in overcoming resistance to implementing green strategies that can enhance and strengthen a sustainable competitive advantage. The organization can be environmentally responsible and competitive in concordance with practical green strategies. By focusing on sustainability as an objective, organizations made radical modifications in their strategic marketing policies to pursue a holistic green marketing orientation and in return, achieved superior business performance.

## Limitations and future research

The present study has certain limitations. First, the data were collected from a specific sector, i.e., the pharmaceutical manufacturing industry whereby other sectors have more negative environmental influences, therefore other studies should consider different types of firms and also employ comparative results between different industries or among different countries.

Second, the relationship between the green marketing orientation and sustainable competitive advantage requires more thorough investigation as the significance of SCA and impacts cannot be fully covered in a single study; rather this requires more attention to different aspects, e.g., drivers of SCA (Papadas et al., 2019) and their role in implementing GMO in organizations.

Similarly, further future studies are needed to explore the moderating effect of tactical, short-term GM practices over the GM strategy-performance relationship. It may serve as “fine-tuning” of the core long-term green marketing strategy. The drivers of GMO (such as competitive environment) in the context of developing countries would be a worthwhile and impactful study.

Finally, future studies should measure holistic GMO with longitudinal data and compare the results before and after opting for the GMO.

## Data availability statement

The original contributions presented in the study are included in the article/Supplementary material, further inquiries can be directed to the corresponding author/s.

## Author contributions

FS has done this work under the supervision of JM as a part of her PhD dissertation. Both authors contributed to the article, the first author worked under the guidance of latter and he continuously guided, reviewed the work and helps in the correction and finalizing the final draft. Both authors approved the final draft.

## Funding

This research was funded by National Natural Science Foundation of China (Grant Nos. 71932007 and 72172119).

## Conflict of interest

The authors declare that the research was conducted in the absence of any commercial or financial relationships that could be construed as a potential conflict of interest.

## Publisher's note

All claims expressed in this article are solely those of the authors and do not necessarily represent those of their affiliated organizations, or those of the publisher, the editors and the reviewers. Any product that may be evaluated in this article, or claim that may be made by its manufacturer, is not guaranteed or endorsed by the publisher.
