# Supplementary material for: Green marketing orientation impact on business performance: Case of pharmaceutical industry of Pakistan
Source: Front Psychol. 2022 Sep 27;13:940278. doi: 10.3389/fpsyg.2022.940278 (PMC9552945; doi:10.3389/fpsyg.2022.940278)
Supplement: Supplementary file 3 [file Table_3.docx]

**Research Questionnaire (Ph.D. Research Study)**

我是一名管理科学的博士研究生，正在进行一项关于绿色营销实践及其在组织中的实施的调查。我非常重视您的合作，谨请您对以下问题作出回应。此次调查是由不同类型的问题组成，且整个问卷中涉及的问题没有对错之分，请您真实反映您的个人意见。本次调查只做学术研究，您提供的问卷数据我们会严格保密。谢谢合作！

I am a PhD scholar of Management Sciences, conducting a survey on **“Green Marketing Practices”** and their implementation in the organizations**.** You are kindly requested to respond to the following statements. I therefore value your cooperation very highly. The survey comprises of different kind of questions. There is no right or wrong answer to the question. I am only interested in your personal opinions. Your responses will be treated in strict confidence and will only be used for research purposes. Thank you in advance for your kind cooperation.

**Section A: General Information (基本信息)**

| **指定/ Designation:** |  | **男性/女性:** | |  |
| --- | --- | --- | --- | --- |
| **工作经验 /Work Experience:** |  | | **行业:** |  |
| **年龄/ Age:** |  | **公司名称:** | |  |

| **请通过以下方式分享您在您的组织中的企业社会责任实践经验:(请圈出一个选项)**  **Please share your experience about Corporate Social Responsibility practices in your organization by rating the following:**  ***(Please circle one option only)*** | **Strongly Disagree** | **Disagree** | **Neutral** | **Agree** | **Strongly Agree** |
| --- | --- | --- | --- | --- | --- |
| 我们组织提供的产品或服务的质量优于竞争对手的产品或服务。  The quality of the products or services that our organization offers is better than that of the competitor's products or services. | **1** | **2** | **3** | **4** | **5** |
| 我们的组织实施了一些特殊的计划来减少它对自然环境的负面影响。  Our organization implements special programs to minimize its negative impact on the natural environment. | **1** | **2** | **3** | **4** | **5** |
| 我们的组织鼓励员工参加志愿活动  Our organization encourage its employees to participate in voluntarily activities. | **1** | **2** | **3** | **4** | **5** |
| 我们的组织致力于促进社会福利的运动和项目。  Our organization contributes to campaigns and projects that promote the well-being of the society. | **1** | **2** | **3** | **4** | **5** |
| 我们的织支持在问题地区工作的非政府组织。  Our organization supports non-governmental organizations working in problematic areas. | **1** | **2** | **3** | **4** | **5** |
| 我们的组织为子孙后代创造美好生活投入资金  Our organization makes investment to create a better life for future generations. | **1** | **2** | **3** | **4** | **5** |
| 我们的组织以考虑到后代的可持续增长为目标。  Our organization targets sustainable growth which considers future generations. | **1** | **2** | **3** | **4** | **5** |
| 请说明贵组织的竞争环境  **Kindly indicate about Competitive Environment for your organization:** | | | | | |
| 我们行业的竞争强度很高  The competitive intensity in our industry is high | **1** | **2** | **3** | **4** | **5** |
| 我们的产品在市场上有许多替代品  There are many substitutes in the market for our products. | **1** | **2** | **3** | **4** | **5** |
| **请根据您对贵组织环境文化的认识，对下列各项进行评分:(请圈出一个选项**  **Please rate following according to your perception about environmental culture in your organization:**  ***(Please circle one option only)*** | | | | | |
| 环境问题与贵组织组织的主要职能密切相关.  Environmental issues are very relevant to the major function of our organization. | **1** | **2** | **3** | **4** | **5** |
| 在我们的组织里，我们齐心协力让每一位员工都明白保护环境的重要性.  At our organization, we make a concerted effort to make every employee understand the importance of environmental preservation. | **1** | **2** | **3** | **4** | **5** |
| 我们努力推动环境保护成为各部门的主要目标。  We try to promote environmental preservation as a major goal across all departments. | **1** | **2** | **3** | **4** | **5** |
| 我们的组织有一份明确的政策声明，敦督促各运营领域都提高环保意识  Our organization has a clear policy statement urging environmental awareness in every area of operations | **1** | **2** | **3** | **4** | **5** |
| 保护环境是本组织的一项高度优先的活动。  Environmental preservation is a high priority activity in our organization. | **1** | **2** | **3** | **4** | **5** |
| 保护环境是我们组织的核心价值观  Preserving the environment is a central corporate value in our organization. | **1** | **2** | **3** | **4** | **5** |
| **通过以下问题来表明您所在组织的绿色营销实践: (请圈出一个选项)**  **Indicate about practices of green marketing in your organization by rating following questions:**  ***(Please circle one option only)*** | | | | | |
| 我们组织想要提高工厂的质量绩效。  Our organization wants to improve the plant’s quality performance. | **1** | **2** | **3** | **4** | **5** |
| 我们的组织想要改善工厂的规章制度。  Our organization wants to improve the plant’s regulatory compliance. | **1** | **2** | **3** | **4** | **5** |
| 我们组织想要改善工厂的形象。  Our organization wants to improve the plant’s image. | **1** | **2** | **3** | **4** | **5** |
| **战略性绿色营销导向是长期的、聚焦企业环境战略的最高管理者行动和政策。绿色营销定位是长期的，最高管理层的行动和政策具体集中在企业环境战略。(请圈出一个选项)**  **Strategic green marketing orientation is long-term, top management actions and policies speciﬁcally focusing on corporate environmental strategy.**  ***(Please circle one option only)*** | | | | | |
| 我们在生产过程中投资低碳技术。  We invest in low-carbon technologies for our production processes. | **1** | **2** | **3** | **4** | **5** |
| 我们采用特定的环境政策来选择合作伙伴。  We use speciﬁc environmental policy for selecting our partners. | **1** | **2** | **3** | **4** | **5** |
| 我们投资于研究和开发项目，以创造环境友好型产品/服务。  We invest in R&D programs in order to create environmentally friendly products/services. | **1** | **2** | **3** | **4** | **5** |
| 我们努力在产品/服务中使用可再生能源。  We make efforts to use renewable energy sources for our products/services. | **1** | **2** | **3** | **4** | **5** |
| 我们的组织建立了一个专门研究环境问题的独立部门  We have created a separate department/unit specializing in environmental issues for our organization. | **1** | **2** | **3** | **4** | **5** |
| 我们与利益相关者就本组织的环境方面进行对话。  We engage in dialogue with our stakeholders about environmental aspect of our organization. | **1** | **2** | **3** | **4** | **5** |
| 我们参与环保商业网络。  We participate in environmental business networks. | **1** | **2** | **3** | **4** | **5** |
| 我们进行市场调查以发现市场中的绿色需求。  We implement market research to detect green needs in the marketplace. | **1** | **2** | **3** | **4** | **5** |
| 在其他目标市场中，我们也瞄准有环保意识的消费者。  Among other target markets, we also target to environmentally-conscious consumers. | **1** | **2** | **3** | **4** | **5** |
| **策略性的绿色营销导向是指将传统的营销组合转变为绿色营销组合的短期行动。。(请圈出一个选项)**  **Tactical green marketing orientation is involving short-term actions that transform the traditional marketing mix into a greener one.**  ***(Please circle one option only)*** | | | | | |
| 我们鼓励使用电子商务，因为它更环保。  We encourage the use of e-commerce, because it is more eco-friendly. | **1** | **2** | **3** | **4** | **5** |
| 我们更喜欢用数字通讯的方式来宣传我们的产品，因为它更环保。  We prefer digital communication methods for promoting our products/services, because it is more eco-friendly. | **1** | **2** | **3** | **4** | **5** |
| 我们在产品/服务中使用可回收或可重复使用的材料。  We use recycled or reusable materials in our products/services. | **1** | **2** | **3** | **4** | **5** |
| 我们在采购过程中尽可能采用无纸化政策。  We apply a paperless policy in our procurement where possible. | **1** | **2** | **3** | **4** | **5** |
| 我们承担了环保产品/服务的额外成本。  We absorb the extra cost of an environmental product/service. | **1** | **2** | **3** | **4** | **5** |
| **内部绿色营销导向是指一个组织的员工和员工对环境意识的实践和行动。(请圈出一个选项)**  **Internal green marketing orientation refers to the practices and actions towards environmental awareness among employees and staff of an organization.**  ***(Please circle one option only)*** | | | | | |
| 模范的环境行为在我们的组织中得到认可和奖励。  Exemplar environmental behavior is acknowledged and rewarded in our organization. | **1** | **2** | **3** | **4** | **5** |
| 候选人的环保活动是我们招聘过程中的额加分项。  Environmental activities by candidates are a bonus in our recruitment process. | **1** | **2** | **3** | **4** | **5** |
| 我们设立了促进环保行为的内部环境奖竞赛。  We have created internal environmental prize competitions that promote eco-friendly behavior. | **1** | **2** | **3** | **4** | **5** |
| 我们成立环保委员会，对环境绩效进行内部审核。  We form environmental committees for implementing internal audits of environmental performance. | **1** | **2** | **3** | **4** | **5** |
| 我们为员工组织演示，让他们了解我们的绿色营销战略。  We organize presentations for our employees to inform them about our green marketing strategy. | **1** | **2** | **3** | **4** | **5** |
| 我们鼓励员工使用环保产品/服务。  We encourage our employees to use eco-friendly products/services. | **1** | **2** | **3** | **4** | **5** |
| 我们的员工认可我们组织的环境价值观。  Our employees believe in the environmental values of our organization. | **1** | **2** | **3** | **4** | **5** |
| **可持续竞争优势是指公司资产、特质或难以复制或超越的能力或能力难以复制或超越;并形成了优于竞争对手的长期有利地位。(请圈出一个选项)**  **Sustainable competitive advantages** are company assets, attributes, or abilities that are difficult to duplicate or exceed; and provide a superior or favorable long term position over competitors.  ***(Please circle one option only)*** | | | | | |
| 我们的组织比竞争对手更有能力进行研发。  Our organization is more capable of R&D than the competitors. | **1** | **2** | **3** | **4** | **5** |
| 我们组织的管理能力比竞争对手强。  Our organization has better managerial capability than the competitors. | **1** | **2** | **3** | **4** | **5** |
| 我们的盈利能力更好了。  Our proﬁtability is better. | **1** | **2** | **3** | **4** | **5** |
| 我们组织的企业形象比竞争对手更好。  Τhe corporate image of our organization is better than that of the competitors. | **1** | **2** | **3** | **4** | **5** |
| 竞争对手很难代替我们组织的竞争优势。  Τhe competitors are difficult to take the place of our organization competitive advantage. | **1** | **2** | **3** | **4** | **5** |
| **生态创新是指减少环境影响的创新产品和过程。(请圈出一个选项)**  **ECO-Innovation** refers to [innovative](https://en.wikipedia.org/wiki/Innovative) products and processes that reduce environmental impacts.  ***(Please circle one option only)*** | | | | | |
| 我们正在用环境友好的投入资源来替代有毒物质。  We are substituting toxic inputs with environmentally friendly. | **1** | **2** | **3** | **4** | **5** |
| 我们正在减少投入，以尽量减少环境风险和影响  We are using fewer inputs to minimize the environmental risks and impacts. | **1** | **2** | **3** | **4** | **5** |
| 我们正在从“肮脏”技术转向更清洁的技术。  We are switching from "dirty" to cleaner technologies. | **1** | **2** | **3** | **4** | **5** |
| 我们正在对投入物、材料和废物进行内部回收。  We are doing internal recycling of inputs, materials and wastes. | **1** | **2** | **3** | **4** | **5** |
| **相对于业务上的主要竞争对手，请对以下各项进行评级，以表明贵组织的经营业绩。:(请圈出一个选项)**  ***Relative to business major competitors, rate the following to indication the business performance of your organization:***  ***(Please circle one option only)*** | | | | | |
| 我们的服务质量比竞争对手的好得多。  The quality of our service is much better than competitors. | **1** | **2** | **3** | **4** | **5** |
| 我们的销售人员的效率比我们的竞争对手高得多。  Our sale force effectiveness is much better than our competitors. | **1** | **2** | **3** | **4** | **5** |
| 我们的产品比竞争对手的质量好  Our image for quality is better than our competitors. | **1** | **2** | **3** | **4** | **5** |
| 我们公司的议价能力优于我们的主要竞争对手  Our bargaining position of our company is better than our major competitors. | **1** | **2** | **3** | **4** | **5** |
| 我们获得市场份额的组织能力很强。  Our organization ability to gain market share is strong. | **1** | **2** | **3** | **4** | **5** |
| 我们组织的规模经济胜过我们的竞争对手。  Our organization’s economies of scale are better than our competitors. | **1** | **2** | **3** | **4** | **5** |
| 与竞争对手相比，我们组织的税前利润很高。  Our organization pre-tax profitability is high as compared to competitors. | **1** | **2** | **3** | **4** | **5** |
| 我们组织的市场占有率比竞争对手高。  Our organization market share is high as compared to competitors. | **1** | **2** | **3** | **4** | **5** |
| 我们组织的公众环境形象较好  The public environmental image of our organization is better. | **1** | **2** | **3** | **4** | **5** |
| **通过回答以下问题来表明你的组织承受了多大的环境压力。(请圈出一个选项)**  **Indicate how much environmental pressure is beard by your organization by answering following question.**  ***(Please circle one option only)*** | | | | | |
| 我们的行业是全国监管最严格的行业之一。  Our industry is one of the most highly regulated industries in the country. | **1** | **2** | **3** | **4** | **5** |
| 我们的组织在环境活动方面有很大的自由。  Our organization is allowed a great deal of latitude in environmental activities. | **1** | **2** | **3** | **4** | **5** |
| 我们的组织经常与环境保护局发生争执。  Our organization is constantly at odds with EPA. | **1** | **2** | **3** | **4** | **5** |
| 我们的组织经常受到环境监管官员的检查。  Our organization is subject to frequent inspections by environmental regulatory officials. | **1** | **2** | **3** | **4** | **5** |
| 我们组织的环境问题相对较少。  Our organization have relatively few environmental problems. | **1** | **2** | **3** | **4** | **5** |
| 我们这个行业的一些成员经常受到环保局严厉的罚款和处罚的威胁。  Our industry has members who are often threatened with stiff EPA regulatory fines and penalties. | **1** | **2** | **3** | **4** | **5** |
| **市场不确定性: 表明你对这些描述您组织的市场环境的同意程度(请圈出一个选项)**  **Market Uncertainty**: Indicate your degree of agreement about how well these statements describe the market environment for your organization.  ***(Please circle one option only)*** | | | | | |
| 市场上竞争对手的活动很不确定。  Competitor activities in the market are quite uncertain. | **1** | **2** | **3** | **4** | **5** |
| 我们的产品市场上有许多新的竞争者。  Our product-market has many new competitors. | **1** | **2** | **3** | **4** | **5** |
| 我们的产品在市场上的竞争情况是很难预测的。  Our product-market competitive conditions are highly unpredictable. | **1** | **2** | **3** | **4** | **5** |
| 我们顾客的喜好变化很快。  Our Customers’ preferences change quite rapidly. | **1** | **2** | **3** | **4** | **5** |
| 我们这个行业的客户需求变化很快。  Customers’’ needs in our industry are changing quite rapidly. | **1** | **2** | **3** | **4** | **5** |
